# First Reported Case of a Clinically Nonresponsive-to-Itraconazole *Alternaria alternata* Isolated from a Skin Infection of a Nonimmunocompromised Patient from Romania

**DOI:** 10.3390/jof9080839

**Published:** 2023-08-11

**Authors:** Ioana Alina Colosi, Maria Crișan, Dan Alexandru Țoc, Horațiu Alexandru Colosi, Carmen Georgiu, Marcela Sabou, Carmen Costache

**Affiliations:** 1 Microbiology Department, Iuliu Hatieganu University of Medicine and Pharmacy, 400012 Cluj-Napoca, Romania; 2 Histology Department, Iuliu Hatieganu University of Medicine and Pharmacy, 400012 Cluj-Napoca, Romania; 3 Cluj County Emergency Hospital, 400000 Cluj-Napoca, Romania; 4 Division of Medical Informatics and Biostatistics, Department of Medical Education, Iuliu Hatieganu University of Medicine and Pharmacy, 400349 Cluj-Napoca, Romania; 5 Pathological Anatomy Department, Iuliu Hatieganu University of Medicine and Pharmacy, 400006 Cluj-Napoca, Romania; 6 Institut de Parasitologie et de Pathologie Tropicale, UR7292 Dynamique des Interactions Hôte Pathogène, Fédération de Médecine Translationnelle, Université de Strasbourg, F-6700 Strasbourg, France; 7 Laboratoire de Parasitologie et Mycologie Médicale, Les Hôpitaux Universitaires de Strasbourg, F-6700 Strasbourg, France

**Keywords:** *Alternaria alternata*, fungal infection, skin ulceration, antifungal resistance, susceptibility testing

## Abstract

Background: *Alternaria alternata* is a melanic fungus capable of causing a wide variety of infections, some of which have lethal potential. It is a ubiquitous fungus and a well-known plant pathogen. Cutaneous infections with *Alternaria alternata* most often occur in the extremities of patients who perform conventional agriculture, thus being exposed to occupational hazards leading to the disruption of the skin barrier. Methods: This paper presents the first case report from Romania of an itraconazole nonresponsive cutaneous alternariosis in a patient without any type of immunosuppression. Results: After an initial misdiagnosis regarding the etiology of the patient’s skin infection, two successive punch biopsies, followed by mycologic examination, lead to the final diagnosis of cutaneous alternariosis. Treatment guided by antifungal susceptibility testing has been instituted, leading to the gradual healing of the patient’s skin ulcerations. Conclusions: The ability of *Alternaria alternata* to infect immunocompetent human hosts and to develop resistance to antifungal drugs highlight the importance of correctly diagnosing the etiology of skin ulcerations and instituting appropriate treatment guided by antifungal susceptibility testing whenever the suspicion of a fungal skin infection is plausible.

## 1. Introduction

Fungal infections are increasingly taking the spotlight in the field of clinical microbiology. Their clinical relevance has recently been enhanced by the COVID-19 pandemic. From highly resistant strains of *Candida auris* to COVID-19-associated pulmonary aspergillosis (CAPA) or mixed etiology CAPA, there is little doubt that we are facing an overwhelming pressure on many clinical settings caused by various fungal infections. Mycoses gradually become more difficult to treat and, similar to bacteria, some of the circulating fungal strains are on the verge of panresistance [1,2,3].

A fungus recently described as the etiologic agent of several infections is *Alternaria alternata*. This melanic fungus is part of the order Pleosporales, class Dothideomycetes, subphylum Pezizomycotina, and phylum Ascomycota. It is a ubiquitous fungus, its airborne spores being found in soil, water, on objects, and on decaying organic material. Alternaria alternata is recognized as a major plant pathogen. Known as a saprophytic and opportunistic mold, its involvement in clinical infections in patients with a compromised immune system is not surprising. However, recent case reports also highlight the presence of *Alternaria alternata* infections in patients without any type of immunosuppression [4,5,6].

There is a wide variety of infections produced by *Alternaria alternata*. Usually, skin infections produced by these types of opportunistic fungi involve the existence of a discontinuity of the skin. An intact skin barrier is able to protect the human organism from dehydration, all types of environmental damage, as well as infections. Thus, a breach in this barrier may lead to skin infections. It is therefore not surprising that these infections are usually present in people using conventional methods of agriculture, such as farm laborers or harvesters [7].

*Alternaria alternata* possesses many virulence factors like its unusually thick cell wall, toxins, proteases, enzymes, reactive oxygen species (ROS), and even allergenic proteins. *Alternaria alternata* produces a variety of toxic metabolites, including mycotoxins. However, not all have proven involvement in human infections and more studies in this area are needed for better understating of this pathogen. One of the most well-known toxins produced by this fungus is alternariol, which has been shown to have cytotoxic and genotoxic effects on mammalian cells. They can affect the cell structure, induce DNA damage, and contribute to tissue injury and inflammation during infection [7,8,9].

There are several proteolytic enzymes produced by this fungus that may interfere with the infection process. Some of these are serine proteases, metalloproteases, and aspartic proteases. These enzymes can degrade host proteins and contribute to tissue invasion and immune evasion by breaking down the extracellular matrix components and some immune system proteins. A particular group of enzymes with an important role in plant infections is cell-wall-degrading enzymes like cellulases and pectinases. These enzymes are important for breaking down components of the plant cell wall and can potentially be involved in tissue invasion. In plants, they are also important for the fungus to gain access to nutrients and thus successfully overcome the physical barriers and promote infection [7,8,9].

*Alternaria alternata* is also able to produce a wide range of allergenic proteins that can trigger allergic responses in susceptible individuals. These proteins are primarily associated with respiratory allergies, asthma, and allergic rhinitis. When inhaled, they can stimulate immune cells, leading to allergic inflammation and symptoms such as wheezing, coughing, and nasal congestion. This fungus can also generate ROS, including superoxide radicals and hydrogen peroxide. These ROS can induce oxidative stress in host tissues by causing damage to lipids, proteins, and DNA. Oxidative stress contributes to tissue damage, inflammation, and host immune response modulation during infection. Fungal melanin is probably the best characterized virulence factor of this microorganism. Melanin protects the fungus from environmental stresses, such as UV radiation and reactive oxygen species (ROS). It also acts like an antiphagocytic agent, and due to its chemical structure, it is able to deactivate ROS [7,8,9]. Nevertheless, most of the pathogenicity factors listed in the literature were investigated in plant samples and in vitro studies, but little evidence has been published so far on how these factors affect human cells.

The aim of this paper is to present the first reported case in Romania of a cutaneous infection produced by an *Alternaria alternata* strain that was nonresponsive to itraconazole in a patient without immunosuppression.

## 2. Case Report

**Figure 1:**
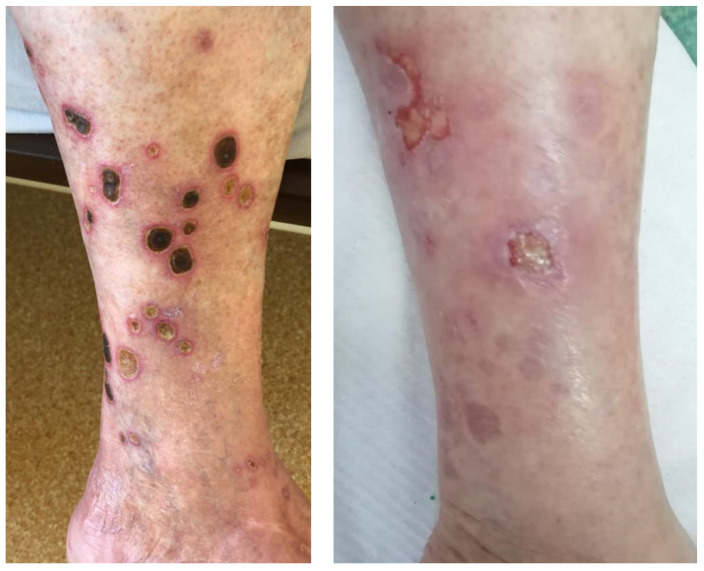
The initial presentation (**left**): several ulcerative lesions of the left calf with black, adherent crusts. After the antifungal treatment (**right**): scars and few remaining ulcerative lesions in the process of healing.

A 75-year-old male patient, with no significant medical history, was admitted in the dermatology department of Cluj County Emergency Hospital with the main complaint of pain and swelling due to several ulcerative lesions on the left calf that were unsuccessfully treated in the outpatient clinic. The physical examination confirmed the presence of several circular ulcerative lesions, with a diameter ranging from 2 cm to 4 cm, covered with black, adherent crusts (Figure 1, left).

Initial laboratory findings were all within the normal range. The wound sample that was collected and sent to the hospital laboratory was positive for *Staphylococcus epidermidis* and *Candida albicans*. An initial diagnosis of ecthyma was proposed, and based on the susceptibility profiles of the isolated strains, the patient received treatment with amoxicillin and fluconazole.

**Figure 2:**
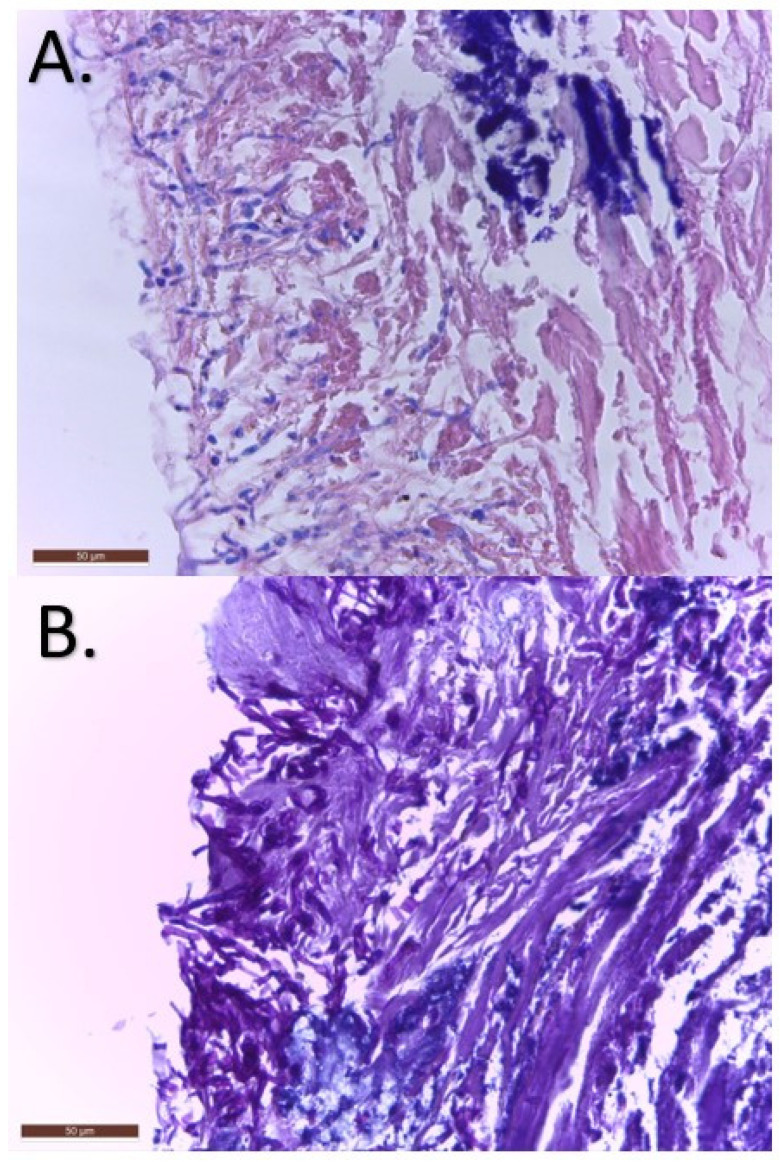
Histopathologic findings of the punch biopsy: septate fungal filaments in hematoxilin–eosine stain (**A**) that were also PAS positive (**B**). The scale bar is set at 50 μm.

Two weeks later, the patient’s condition had not improved and a punch biopsy of 0.4 × 0.4 × 0.5 cm was collected and sent to the hospital pathology laboratory. The results showed an extensive ulceration of the epidermis with inflammatory exudates and septate filaments with 45° angled ramifications that were positive in PAS stain. Also, perivascular inflammation was described in the dermis and hypodermis (Figure 2). A second diagnosis of cutaneous mycosis was proposed, and local as well as systemic treatment with itraconazole 200 mg/day was initiated.

**Figure 3:**
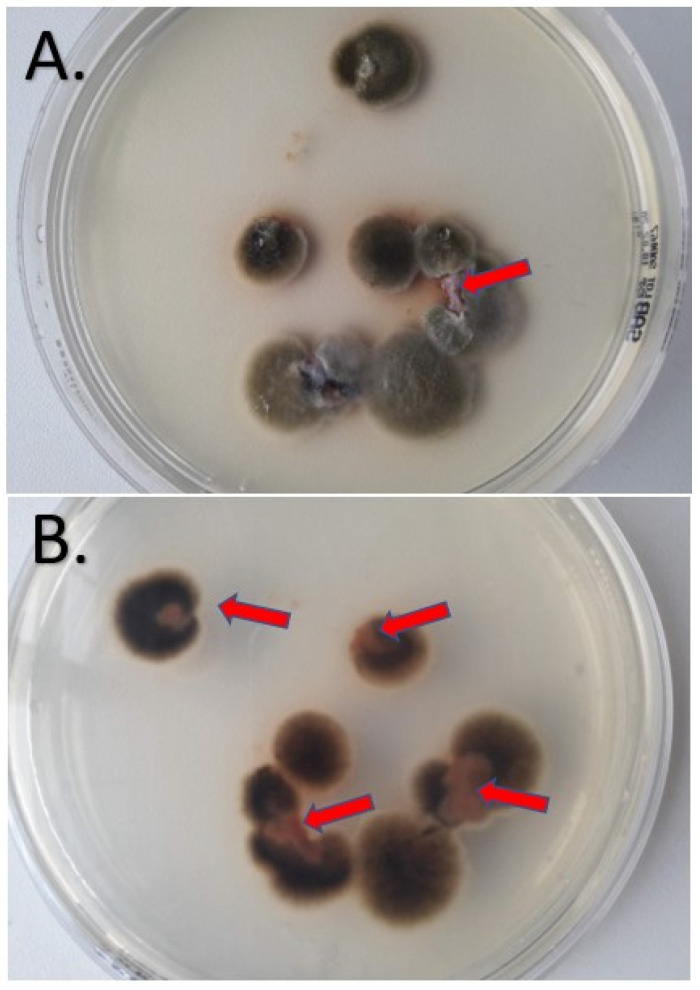
Macroscopic appearance of the *Alternaria alternata* culture from front (**A**) and back (**B**) after 5 days of incubation at 25 °C. The red arrows indicate the biopsy fragments that were inoculated.

At one-month follow-up, the ulcerative lesions had not showed an improvement and a second biopsy was performed in order to determine the fungal etiology. The specimen was cultivated on Sabouraud Chloramphenicol Gentamicin agar (Bio-Rad, Marnes-la-Coquette, France) at 25 °C ± 2 °C and at 35 °C ± 2 °C. After five days, several flat colonies were present with an average size around 5 cm. They presented a floccose texture and dark green color. On the reverse, a black pigment was present. The macroscopic appearance of the fungal colonies is presented in Figure 3.

**Figure 4:**
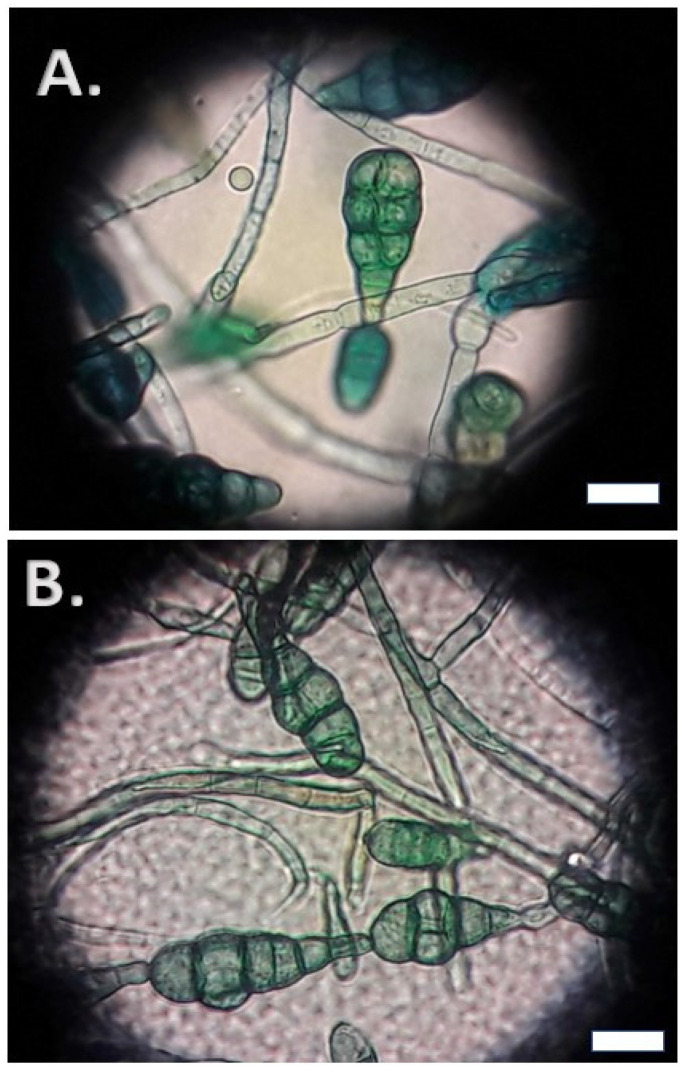
Microscopic appearance of *Alternaria alteranata*. Conidia with transverse and longitudinal septa (**A**), around 25 μm, organized in chains (**B**). The scale bar is set at 10μm.

Cotton blue stain was performed and showed chains of club-shaped conidia with transverse and longitudinal septa. The size of the conidia ranged between 20 and 30 μm and they presented a terminal beak at the distal end. Figure 4 presents the microscopic characteristics of these conidia in cotton blue stain.

Based on the above macroscopic characteristics of the culture and the microscopic features, a diagnosis of alternariosis was proposed. The species identification was performed by sequencing the internal transcribed spacer (ITS) region of the ribosomal RNA using primers ITS1 and ITS4 (Eurofins Genomics GmbH [10]. The obtained sequences, compared to those in the GenBank and Westerdijk Fungal Biodiversity Institute databases, confirmed the species of *Alternaria alternata,* and the final diagnosis of cutaneous infection with *Alternaria alternata* was established. Antifungal susceptibility testing for this strain was conducted using the E-test method according to the manufacturer’s instructions. The E-test MIC (minimal inhibitory concentration) was the lowest drug concentration at which the border of the elliptical inhibition intercepted the scale on the antifungal strip [11]. E-tests (bioMérieux SA, Marcy-l’Étoile, France) were placed on the inoculated RPMI 1640, supplemented with 2% glucose medium plates, and incubated at 35 °C for 24–48 h. This analysis showed that the isolated strain of *Alternaria alternata* presented an elevated MIC (2 mg/L) for itraconazole. Based on these results and on the availability of other treatment options, the treatment was changed to terbinafin orally and miconazole locally. At 5 months follow-up, the lesions were healing successfully, with smooth scars (Figure 1, right).

## 3. Discussion

*Alternaria alternata* is one of the pathogens that have attracted attention in mycology research over the past years. Although its involvement in human infections was up for debate for a while, recent data suggested that *Alternaria alternata* has the potential to cause a wide variety of infections with considerable lethality [7,12]. Hence, when septate fungal filaments are observed during histopathological examination of tissues, other filamentous fungi like *Alternaria* spp. need to be considered in addition to *Aspergillus* spp.

This paper presents the first case report from Romania of a cutaneous alternariosis in a patient without any type of immunosuppression, and only the fourth such case in Europe [6]. Based on lesions localizations, cutaneous infections with *Alternaria alternata* usually occur in the extremities. This underlines the importance of occupational hazards that might lead to the disruption of the skin barrier, leading to infection. Our patient did not report any trauma; however, he declared that he used to perform conventional agriculture at home, thus being exposed to this known risk factor for an infection produced by this fungus.

Although the clinical outcome of our case is positive, healing of those lesions occurred with some scarring. Sometimes, cutaneous fungal infections can lead to scarring, which refers to the formation of fibrous tissue during the wound healing process. Scarring occurs as a result of the body’s attempt to repair the damaged skin. However, in some cases, the scarring can be excessive, leading to functional and cosmetic concerns. Understanding the mechanisms behind post-fungal-infection cutaneous scarring, as well as the psychological implications for affected patients, is crucial for complete and effective management of these cases [13,14].

The development of scars after cutaneous fungal infections is influenced by various factors. The severity and depth of the initial wound, the extent of tissue damage, and the individual’s healing response all play a role. Fungal infections can cause chronic inflammation and delayed wound healing. In addition, the presence of fungal elements in the wound can trigger an exaggerated immune response, leading to increased collagen production [15].

Scarring resulting from cutaneous fungal infections can have a significant psychological impact on affected patients. The visibility of scars can affect body image and self-esteem, leading to decreased confidence and social withdrawal. Patients may experience deep feelings of embarrassment, anxiety, and depression. Psychological distress related to scarring can have a huge impact on the quality of life and should be consistently addressed [16,17].

There are many available treatments that address both the physical and psychological aspects of scarring. Some approaches include the use of topical treatments, laser therapy, surgery, and some scar management techniques. Various cosmetic products containing ingredients such as silicone, corticosteroids, or vitamin E can be applied to the scarred area to improve its appearance and texture. These topical treatments aim to hydrate the skin, reduce inflammation, and promote collagen remodeling [14,15].

Laser treatments can help minimize scar visibility and improve skin texture. These procedures stimulate collagen production, reduce redness, and promote smoother skin. In cases of hypertrophic scars, surgical procedures like scar revision or scar excision may be considered. These procedures involve modifying the scar tissue to improve its appearance. An alternative is to remove the scar entirely. Surgical interventions are typically reserved for more severe, hypertrophic, or persistent scars. Other options like scar massage and silicone gel sheets are commonly used to manage scars. These techniques may help soften the scar tissue, flatten raised scars, and reduce itching or discomfort [14,15].

Psychological support and counseling are crucial for individuals affected by scarring. Therapies such as cognitive-behavioral therapy (CBT) or acceptance and commitment therapy (ACT) can help individuals cope with the psychological impact of scarring, improve body image, and enhance self-esteem. Support groups or online communities can provide a platform for individuals to share experiences and receive emotional support from others facing similar challenges [18].

Cutaneous alternariosis has been extensively reviewed by Hu et al., who included 29 case reports [12]. However, out of those 29 cases, only 2 came from immunocompetent patients. The rest of the cases described cutaneous alternariosis in patients with different levels of immunosuppression, mostly after an organ transplant (19/29 cases) like renal, cardiac, lung or liver. Also, as previously mentioned, this cutaneous infection targeted primarily the extremities, mostly lower and upper limbs. The clinical appearance of these cases was diverse, including papules, nodules, lacerations, and even ulcers. The diagnosis to the species level was performed in 20/29 cases and *Alternaria alternata* was the most common etiology, with only 1 other case of *Alternaria arborescens* being included. The treatment of these infections included mostly itraconazole or voriconazole in different regimes. Only three cases were described as fatal [12].

The niche of cutaneous infections produced by *Alternaria* spp. in immunocompetent patients was recently tackled by Jebari et al. in a 2022 article which described a case of cutaneous alternariosis and included 10 case reports in order to provide a brief review [6]. Only six cases were diagnosed at the species level, and five out of six infections were produced by *Alternaria alternata*. Itraconazole was also the main antifungal used for the treatment of these infections. Other alternative options described in these case reports were amphotericin B, fluconazole, and econazole in different combinations. The outcome was favorable in 9/10 cases, and the only 1 that resulted in death was in a patient who received corticosteroids. However, in two cases, the patients received a topical steroid treatment with a favorable outcome. Thus, the hypothesis emerges that perhaps previous corticosteroid therapy in cutaneous alternariosis poses an important role, but future studies should address this issue.

Itraconazole is an antifungal drug in the azole family that acts by disrupting the fungal cell membrane synthesis. Specifically, it inhibits an enzyme called 14-α-demethylase that is involved in the conversion of lanosterol to ergosterol. Resistance to itraconazole was described in yeasts and filamentous fungi. In *Candida* spp., resistance to itraconazole has been attributed either to a point mutation in the *ERG11* gene responsible for 14-α-demethylase synthesis, or to the presence of efflux pumps like Cdr1 and Cdr2. Regarding the filamentous fungi, in *Aspergillus* spp. other resistance mechanisms have also been described, like mutations in the *CYP51A* gene or TR_34_/L98H, which is responsible for a specific phenotype, resistant to all azoles. A different mechanism involves changes in the ergosterol biosynthesis pathway, or the expression of alternative pathways that can bypass the target enzyme inhibited by azoles. This can allow the fungus to maintain sufficient ergosterol levels in the cell membrane despite azole treatment. Other mechanisms have also been described, but with lower frequency [19,20]. Azole resistance in *Alternaria alternata* has been reported in both clinical isolates causing human infections and in agricultural settings where azole fungicides are extensively used to control plant diseases. The emergence of azole-resistant strains poses challenges for effective treatment and disease management [21].

Terbinafine acts by inhibiting ergosterol synthesis. It enters the fungal cell and targets the enzyme called squalene epoxidase, responsible for producing squalene epoxide, an important step in the synthesis of ergosterol. Also, by inhibiting that enzyme, squalene levels in the fungal cell increase, leading to the inhibition of other steps in the ergosterol biosynthesis. The depletion of ergosterol in the fungal cell leads to the disruption of the membrane and cellular death [22]. By combining miconazole and terbinafine, we aimed to target the ergosterol synthesis at two levels, thus providing full recovery for the patient.

To our knowledge, the combination of itraconazole and terbinafine was used only once before in the treatment of cutaneous alternariosis, as presented by Minigawa et al. [23]. In their case report, the patient had a debilitating underlying condition, autoimmune hemolytic anemia, and the treatment was ineffective. The presented case report is the first successful treatment with miconazole and terbinafine in an immunocompetent patient. This combination may represent an alternative in the treatment of cutaneous alternariosis caused by strains that are non-responsive to itroconazole in the context of the lack of standardization in these cases. This situation was previously addressed in articles which pleaded for a combination of surgical treatment and antifungal medication in the context of immunosuppression induced by corticosteroid treatment, in patients with cutaneous alternariosis [24,25].

Understanding the underlying mechanisms of azole resistance in *Alternaria alternata* is crucial for developing strategies to overcome or prevent it. This may involve the development of novel antifungal agents, combination therapies, or the use of alternative disease management strategies in agriculture to minimize the selective pressure for resistance. Also, the exact mechanism of infection produced by *Alternaria alternata* is not yet understood. Although several factors have been extensively addressed in plants, in mammalian tissues this fungus may exhibit different patterns of infection. Since cases have started to appear all over the world, it seems necessary to address this issue in the near future.

Nevertheless, azole resistance in *Alternaria* spp. is not well understood. To the best of our knowledge, this case report is the second reported case of cutaneous alternariosis that is nonresponsive to itraconazole, the first being described by Gomes et al., 2011 [26]. Although resistance is rare, antifungal susceptibility testing in *Alternaria* spp. should be routinely performed since the patient’s outcome might be significantly influenced by the result of such an investigation [11,27]. Future studies should also aim to describe the mechanism of antifungal resistance in clinical isolates of *Alternaria* spp. Since this enigmatic pathogen seems to be part of the differential diagnosis in certain cases of cutaneous mycosis, we need to be prepared to successfully face the threat of resistant strains in the future.

## Data Availability

MDPI Research Data Policies.
